# Do the three-dimensional parameters of brace-wearing patients with AIS change when transitioning from standing to sitting position? A preliminary study on Lenke I

**DOI:** 10.1186/s12891-022-05380-z

**Published:** 2022-05-04

**Authors:** Xiaohui Zhang, Daoyang Yang, Shuo Zhang, Jun Wang, Yuan Chen, Xiaoran Dou, Yanan Liu, Xianglan Li, Bagen Liao

**Affiliations:** 1 Department of Sports Medicine, Guangzhou Sport University, 1268 # Guangzhou Avenue, Guangzhou, 515500 Guangdong Province China; 2 Gosun Medical Imaging Diagnosis Center of Guangdong Province, 117 #Liuhua Road, Guangzhou, 515500 Guangdong Province China

**Keywords:** Adolescent idiopathic scoliosis, 3D parameters, Bracing, Sitting position

## Abstract

**Background:**

Bracing is the most common conservative treatment for preventing the progression of adolescent idiopathic scoliosis (AIS) in patients with a curve of 25°–40°. X-ray examinations are traditionally performed in the standing position. However, school-age teenagers may take more time to sit. Thus far, little is known about three-dimensional (3D) correction in the sitting position. Hence, this study aimed to determine the effects of standing and sitting positions on 3D parameters during brace correction.

**Methods:**

We evaluated a single-center cohort of patients receiving conservative treatment for thoracic curvature (32 patients with AIS with a Lenke I curve). The 3D parameters of their standing and sitting positions were analyzed using the EOS imaging system during their first visit and after bracing.

**Results:**

At the patients’ first visit, sagittal plane parameters such as thoracic kyphosis (TK), lumbar lordosis (LL), and sacral slope decreased when transitioning from the standing position to the sitting position (standing 29° ± 6°, 42° ± 8°, and 42° ± 8° vs. sitting 22° ± 5°, 27° ± 6°, and 24° ± 4°; *p* < 0.001), whereas pelvic tilt (PT) increased and sagittal vertical axis shifted forward (standing 9° ± 6° and 1.6 ± 2.7 cm vs. sitting 24° ± 4° and 3.8 ± 2.3 cm; *p* < 0.001). After bracing, TK and LL decreased slightly (from 29° ± 6° and 42° ± 8° to 23° ± 3° and 38° ± 6°; *p* < 0.001), whereas the thoracolumbar junction (TLJ) value increased (from 3° ± 3° to 11° ± 3°; *p* < 0.001). When transitioning to the sitting position, similar characteristics were observed during the first visit, except for a subtle increase in the TLJ and PT values (standing 11° ± 3° and 9° ± 4° vs. sitting 14° ± 3° and 28° ± 4°; *p* < 0.001). Moreover, the coronal and axial parameters at different positions measured at the same time showed no significant change.

**Conclusions:**

In brace-wearing patients with thoracic scoliosis, compensatory sagittal plane straightening may be observed with a slight increase in thoracolumbar kyphosis, particularly when transitioning from the standing position to the sitting position, due to posterior rotation of the pelvis. Our results highlight that sagittal alignment in AIS with brace treatment is not completely analyzed with only standing X-Ray.

**Trial registration:**

The study protocol was registered with the Chinese Clinical Trial Registry (ChiCTR1800018310).

## Introduction

Adolescent idiopathic scoliosis (AIS) is characterized by a three-dimensional (3D) deformity of the spine and trunk. A recent study by Weinstein et al. demonstrated that bracing significantly reduces the progression of the high-risk curve to the surgical threshold in patients with AIS [1]. When using a brace to treat patients with scoliosis, the correction of coronal, transverse, and sagittal parameters is necessary to achieve optimal outcomes. In all patients, including 28% of those with treatment failure, these parameters were traditionally analyzed in the standing position. However, the changes in the parameters measured in different positions remain to be elucidated in brace-wearing patients with AIS.

The parameters associated with a higher risk of curve progression during bracing are generally considered multidimensional; these parameters include the curve type [2], curve magnitude [2], skeletal maturity [3], menarcheal age [4], peak height velocity [4], and brace treatment initiation time [5]. In addition to the aforementioned identifiable factors, brace compliance [6], initial coronal deformity angular ratio [6, 7], and curve flexibility [8] influence the decision-making of physicians, orthotists, and physiotherapists as these may affect the final correction outcome [8, 9].

Recently, several studies have challenged the coronal correction rate based on the impact of 3D analysis of bracing efficacy [10, 11]. An important prospective study by Kwan et al. reported that considering all three-plane spinal parameters is important for the successful treatment of AIS using braces [12]. In another study, the impact of wearing a brace on the sagittal profile was found to be variable, which included the loss of thoracic kyphosis (TK) and lumbar lordosis (LL) [10]. Similarly, the analysis of sagittal alignment from the head to the pelvis showed that the brace further flattens the patient’s back and causes a large compensatory reorientation of the pelvis. Sagittal balance should be included in the planning and evaluation of brace treatment because it may play a role in the treatment outcome [13].

Standing and sitting are the two most commonly used weight-bearing positions. Currently, the reference values for spinal deformity correction are measured using standing X-ray examinations [14–16]. The sagittal spinopelvic alignment may change in different positions [17–19]. However, limited detector size and single-source divergent X-ray beam were used in the conventional radiological examinations performed in the previous studies, which might have led to image magnification and stitching errors. Previous studies have described the 3D reconstruction of EOS stereoradiography [20–23]. According to the aforementioned studies, the 3D parameters obtained from simultaneous frontal and lateral imaging exhibit excellent reliability in standard- and microdose protocols [21, 23]. Hence, the influence of 3D parameters on the success of brace treatment has gradually garnered attention.

School-age teenagers may take more time to sit. Thus far, little is known about the 3D correction of AIS in the sitting position. Therefore, this study sought to determine the effects of the standing and sitting positions on the 3D parameters measured during the brace treatment of patients with thoracic curvature with a risk of progression.

## Materials and methods

### Study design and participants

This prospective observational cohort study enrolled 32 patients with AIS who met the Scoliosis Research Society criteria for bracing between October 2019 and February 2021. Informed consent was obtained from each patient or their legal guardian before recruitment. Inclusion criteria were the age of 10–15 years, a Cobb angle of 25°–40°, AIS classified as Lenke I, skeletal immaturity (defined as 0–3 on the Risser scale), and < 1 year after menarche.

### Study interventions

All patients wore Chêneau-type braces made using molded casting via computer-aided design/manufacturing.

### Data collection

Radiographic examinations using the EOS imaging system were performed at a 7-day interval between each patient’s first visit and immediately after the patients started using the Chêneau-type brace.

The EOS system is a microdose imaging system that can acquire simultaneous posterior–anterior and lateral views in the standing and sitting positions.

To perform spinal 3D modeling on the sterEOS® workstation, the following anatomical landmarks should be visible on both views: the center of vertebra C7, vertebral endplates of the thoracic and lumbar spine, sacral endplate, and bilateral acetabula. To ensure good visibility of these anatomical landmarks without overlapping, the patient should be positioned as follows during the standing-position acquisition. First, the participant is placed in the middle of the cabin in a weight-bearing position with the pelvis in the isocenter of the platform. Then, the fists are placed on the cheek or clavicle so that the patient’s upper arm is at a 45° angle to the body, with the patient looking straight ahead.

**Fig. 1:**
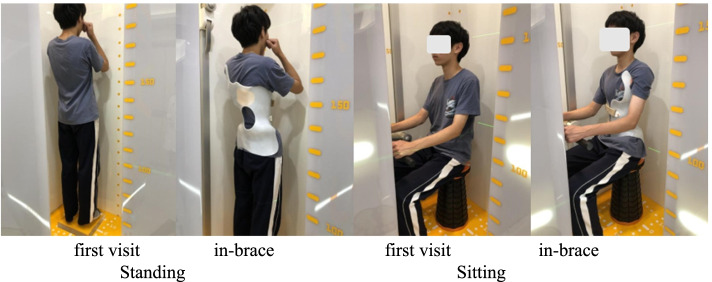
Patient is examined using the EOS imaging system in the standing and sitting positions at the first visit and after bracing (with a Chêneau-type brace)

In the sitting position, patients’ images are obtained as follows [19]. The user should place the patient on a stool with a rounded seat and base to facilitate installation in the EOS. The height of the stool should be adjustable from 40 cm (15 inches) to 55 cm (22 inches) to allow positioning of the femurs parallel to the ground. The crossbar is placed to keep the horizontal line 33 cm from the eye. The patient should place their hands on the bar to imitate the standard sitting position of a student (Fig. 1).

### 3D reconstruction

A semi-automated 3D reconstruction of the spine was performed by a trained and experienced radiologist using a dedicated software with proven results (sterEOS®) [24].

**Fig. 2:**
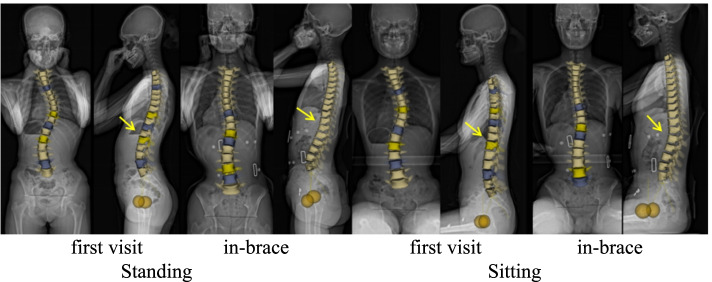
A 13-year-old patient with adolescent idiopathic scoliosis was examined in the standing and sitting positions at the first visit and after bracing. The changes in sagittal plane parameters can been observed (yellow arrow)

The software can identify anatomical points and generate a computerized 3D reconstruction of the complete spine based on the synchronized posterior–anterior and lateral images [23] (Fig. 2). Radiographic parameters were collected along three planes:

1. The sagittal plane—TK (T4–T12), LL (L1–L5), thoracolumbar junction angle (TLJ; T10–L2), pelvic tilt (PT), pelvic incidence, sacral slope (SS), and sagittal vertical axis (SVA)
2. The coronal plane—based on the Cobb angle measurements, the in-brace correction rate was measured using the following equation:
  $$\mathrm{Correction}\;\mathrm{Rate}=\frac{(\mathrm{Prebrace}\;\mathrm{Cobb}\;\mathrm{Angle}-\mathrm{In}-\mathrm{Brace}\;\mathrm{Cobb}\;\mathrm{Angle})}{\mathrm{Prebrace}\;\mathrm{Cobb}\;\mathrm{Angle}}\times100\%$$
  High initial correction of > 50% was of significance for the outcome [7].
3. The transverse plane—the following parameters were used: apical axial vertebral rotation (AVR) [7] and torsion index, which is the average of the two sums of intervertebral axial rotation from the lower junction to the apex and from the apex to the upper junction [25].

### Statistical analysis

Values are expressed as means ± standard deviations (SDs). Statistical analysis was performed using the SPSS software (v. 22.0, SPSS Inc.). The Shapiro–Wilk test was used to test the normality of the distribution of all continuous variables. Normally distributed variables were analyzed using Student’s paired *t*-test; otherwise, the Wilcoxon signed-rank test was used for the comparison. A *p*-value of < 0.05 was considered statistically significant.

## Results

In total, 3 male and 29 female patients with AIS classified as Lenke I were included in the study.

At the first visit, their mean age was 13.4 ± 1.3 (range: 10–14) years. The average Cobb angle of the main curve in the standing position was 36° ± 5°. The correction rate of 28 patients exceeded 50%, and the mean ± SD of the correction rate in these patients was 63% ± 7.56%; in the other 4 patients, the mean ± SD of the correction rate was 44.7% ± 4.25%.

There was no significant difference in the coronal and transverse parameters when transitioning from the standing position to the sitting position at the first visit or after bracing (p > 0.05). Regarding transverse parameters in the standing position, AVR and torsion index measurements decreased after bracing than those measured at the first visit (from 6° ± 1° and 6.58 ± 2.71 to 4° ± 1° and 4.22 ± 2.09; *p* < 0.001). Furthermore, the sagittal parameters TK and LL reduced after bracing (from 29° ± 6° and 42° ± 8° to 23° ± 3° and 38° ± 6°; *p* < 0.01), whereas TLJ increased (from 3° ± 3° to 11° ± 3°; *p* < 0.001). Compared with the corresponding measurements in the sitting position at the first visit, TK, LL, and SS decreased after bracing (from 22° ± 5°, 27° ± 6°, and 27° ± 6° to 15° ± 3°, 22° ± 3°, and 22° ± 4°; *p* < 0.01), whereas TLJ and PT values increased (from 3° ± 2° and 24° ± 4° to 14° ± 3° and 28° ± 4°; *p* < 0.01) (Table 1).

**Table 1:** Three-plane parameters: comparison of values obtained in the standing position versus in the sitting position at the first visit and after bracing

	Standing		*p* Value	Sitting		*p* Value
Parameters	first visit	in-brace		first visit	in-brace	
Sagittal parameters						
TK (°)	29 ± 6	23 ± 3	< 0.001 *	22 ± 5	15 ± 3	< 0.001*
TLJ (°)	3 ± 3	11 ± 3	< 0.001 *	3 ± 2	14 ± 3	< 0.001 *
LL (°)	42 ± 8	38 ± 6	0.010*	27 ± 6	22 ± 3	< 0.001 *
PI (°)	50 ± 6	50 ± 5	0.441	50 ± 7	50 ± 5	0.952
PT (°)	9 ± 6	9 ± 4	0.683	24 ± 4	28 ± 4	< 0.001 *
SS (°)	42 ± 4	40 ± 7	0.168	27 ± 6	22 ± 4	0.010*
SVA (cm)	1.6 ± 2.7	1.7 ± 2.5	0.969	3.8 ± 2.3	3.8 ± 2.1	0.965
Coronal parameters						
Cobb angle (°)	36 ± 5	14 ± 4	< 0.001*	36 ± 5	14 ± 4	< 0.001*
Transverse parameters						
AVR (°)	6 ± 1	4 ± 1	< 0.001*	6 ± 1	4 ± 1	< 0.001*
Torsion index	6.58 ± 2.71	4.22 ± 2.09	< 0.001*	6.55 ± 2.75	4.28 ± 2.16	< 0.001*

^*^ Significant difference (*p* < 0.05)

Compared with the corresponding measurements in the standing position, at the first visit, the sagittal parameters TK, LL, and SS decreased in the sitting position (standing 29° ± 6°, 42° ± 8°, and 42° ± 4° vs. sitting 22° ± 5°, 27° ± 6°, and 24° ± 4°; *p* < 0.001), whereas PT increased (standing 9° ± 6° vs. sitting 24° ± 4°; *p* < 0.001). After bracing, TK, LL, and SS decreased when transitioning from the standing position to the sitting position (from 23° ± 3°, 38° ± 6°, and 40° ± 7° to 15° ± 3°, 22° ± 3°, and 22° ± 4°; *p* < 0.001), whereas TLJ and PT values increased (standing 11° ± 3° and 9° ± 4° vs. sitting 14° ± 3° and 28° ± 4°; *p* < 0.001).

During the first visit and after bracing, a forward shift in SVA was observed when transitioning from the standing position to the sitting position (from 1.6 ± 2.7 cm and 1.7 ± 2.5 cm to 3.8 ± 2.3 cm and 3.8 ± 2.1 cm; *p* < 0.01) (Table 2).

**Table 2:** Three-plane parameters: comparison of values obtained in the standing position versus in the sitting position at the first visit and after bracing

	first visit		*p* Value	in-brace		*p* Value
Parameters	Standing	Sitting		Standing	Sitting	
Sagittal parameters						
TK (°)	29 ± 6	22 ± 5	< 0.001*	23 ± 3	15 ± 3	< 0.001*
TLJ (°)	3 ± 3	3 ± 2	0.893	11 ± 3	14 ± 3	< 0.001*
LL (°)	42 ± 8	27 ± 6	< 0.001*	38 ± 6	22 ± 3	< 0.001*
PI (°)	50 ± 6	50 ± 7	0.968	50 ± 5	50 ± 5	0.281
PT (°)	9 ± 6	24 ± 4	< 0.001*	9 ± 4	28 ± 4	< 0.001*
SS (°)	42 ± 4	27 ± 6	< 0.001*	40 ± 7	22 ± 4	< 0.001*
SVA (cm)	1.6 ± 2.7	3.8 ± 2.3	< 0.001*	1.7 ± 2.5	3.8 ± 2.1	< 0.001*
Coronal parameters			0.386			0.813
Cobb angle (°)	36 ± 5	36 ± 5		14 ± 4	14 ± 4	
Transverse parameters						
AVR (°)	6 ± 1	6 ± 1	0.453	4 ± 1	4 ± 1	0.264
Torsion index	6.58 ± 2.71	6.55 ± 2.75	0.622	4.22 ± 2.09	4.28 ± 2.16	0.131

^*^ Significant difference (*p* < 0.05)

## Discussion

**Fig. 3:**
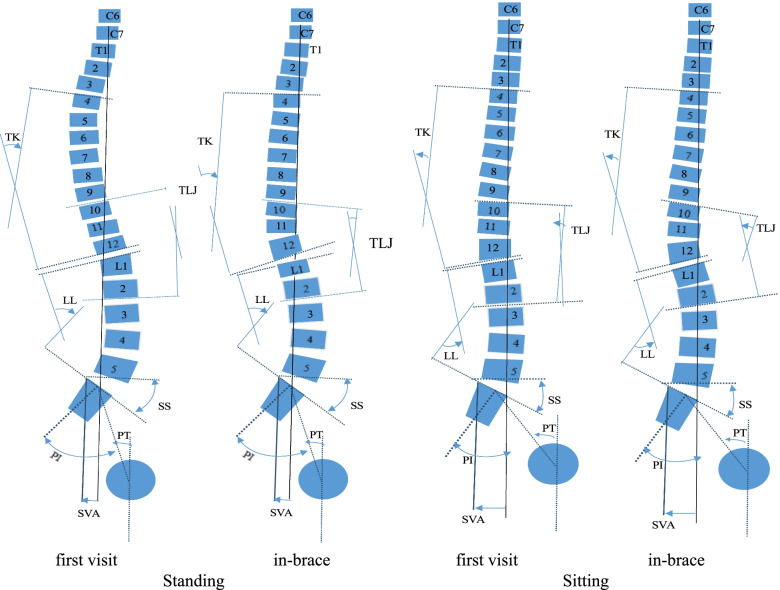
Changes in sagittal plane parameters at the first visit and after bracing

Using 3D reconstruction of the spine, three novel findings relating to the 3D parameters of sitting with bracing were demonstrated. In this study, axial rotation in the apex vertebrae was significantly improved after wearing a brace in two positions—the first finding.Significant differences between the two positions were observed in that the sagittal parameters of bracing were all decreased during the sitting position than during the standing position (Fig. 3)—second finding. Furthermore, no differences in the parameters of the coronal and transverse planes were observed between the two positions, whether at first visit or after bracing—third finding.

The axial-plane parameters and correction of these parameters during bracing are related to the success of brace treatment [11]. In our study, the axial rotation and torsion coefficients of the apical vertebrae improved with brace correction; however, there was no statistically significant difference in them between the standing and sitting positions after bracing. These findings of our study are consistent with those of previous studies reporting that improved axial rotation after bracing suggests that a Chêneau-type brace has a significant effect on AVR compared with a traditional thoracolumbosacral orthopedic brace [26]. This effect may be due to the design of the Chêneau-type brace, which provides detorsion force through the trunk and achieves 3D self-correction of active movement through the release space of the brace. This can be observed during the breathing expansion process [27].

An immediate coronal correction rate of the brace between 30 and 60% may lead to a satisfactory treatment outcome [28–30]. Recently, more studies have focused on the characteristics of the sagittal plane [31, 32]. In our study, the correction of the coronal plane was relatively satisfactory. Changes in the sagittal plane parameters were observed, such as a decrease in TK and LL and an increase in TLJ, which corroborate the results of the study conducted by Courvoisier et al. [24]. Our study confirmed the abovementioned phenomenon in patients with main thoracic curve, where the sitting position was more pronounced than the standing position when the patients wore the brace.

Compared with the standing position, TK, LL, and SS decreased and PT significantly increased in the sitting position at the first visit. Changes in these parameters were similar to those reported in previous studies without brace [18, 19]. The straightening of the spine and associated loss of LL and TK result in a compensatory increase in PT. Notably, as the sacrum becomes more horizontal, SS decreases. Biomechanical studies have demonstrated a correlation between the lumbar spine and sacrum [26, 33–35].

The characteristic of sagittal straightness when standing is described as a “side effect” of brace treatment wherein the trunk follows the brace shape.

The posterior thoracic pad pushes to correct the rib uplift to derotate the apex vertebrae, which may be the cause of the iatrogenic deficiency of TK (T4–T12). In turn, it may result in the straightening of the overall sagittal plane. We found a subtle difference in the increased TLJ (T10–L2) value after bracing, which might be a mechanism of the body’s self-regulation in bracing [10]. The brace exerts corrective force on the lateral and posterior sides of the thoracic and lumbar curves, respectively, affecting the sagittal curves (Fig. 3). The change from the standing position to the sitting position after wearing braces was similar to that noted in sagittal plane characteristics at the first visit, except for an increase in the TLJ value.

To the best of our knowledge, few studies have focused on changes in the parameters measured in the sitting position after wearing a brace. The exclusive use of the standing-position reference values is not comprehensive. In particular, brace-wearing patients with AIS take more time to sit in the weight-bearing position. Our study shows that the sagittal spinopelvic parameters are the most variable in both the standing and sitting positions and may be the primary factors controlling the sagittal plane balance. The spine is straightened, SVA shows positive changes, pelvis rotates backward, and sacrum rests in a more horizontal position when sitting. It is most significant in the pelvic and lower-lumbar segments, followed by the thoracic segment. In children, various segments of the pelvis and spine are interdependent in the standing position [36]. Our data and their interpretation are also consistent with the idea that pelvic positioning determines the overall sagittal balance.

Our novel study also suggests that the influence of the sagittal plane parameters when a patient is seated may be related to the posterior rotation of the pelvis, leading to a straighter sagittal curve and forward shift of the SVA. This effect has also been demonstrated in patients undergoing surgical treatment. When the kyphosis is reduced, the lumbar spine compensates by reducing the lordosis to maintain the coordination of the thoracic and lumbar spine [37]. We showed that after wearing a brace, the whole spine became straight and the SVA moved forward in the sitting position, which was not reported in previous studies.

The effect of maintaining the sitting position for a prolonged time on spinal deformities in adolescents should be studied further. Nonetheless, we believe that the changes in these parameters imply that 3D changes after bracing may not be fully analyzed using standing-only assessments. Furthermore, we infer that the forward movement of the SVA is likely due to a change in the center of gravity that causes the body to move forward to achieve more physiological spinal balance. The reduction of LL and compensatory reduction of TK are the primary changes required to achieve the physiological balance.

A correlation among several segments of the sagittal plane has been demonstrated in surgical studies. An increase in TK following surgery results in an increase in lordosis in the proximal spine [38, 39]. The sagittal plane curvature is mostly hypokyphosis, which is as important as the transverse plane in the overall definition of scoliosis [31]. The segmental analysis of spinal deformity and differences in the structural changes of segmental adaptation mechanism in the related sagittal plane may provide a comprehensive understanding of the sagittal assessment of AIS. The sagittal alignment of the spine in our patients was different from that noted in healthy individuals because of the structural deformity. At the first glance, the curve-type polymorphism of the sagittal plane appears complex.

This study has some limitations. One limitation is the generality of the cross-sectional observation design. Although the cohort comprised patients from all over the country, it does not ensure that they were representative of the entire population of adolescents with AIS. Although only a microdose of radiation was used in the experimental procedure, the procedure itself was not entirely radiation-free. As patients who did not provide consent were excluded, the sample size was small, which is another limitation of this study. Moreover, the lack of patient-reported outcomes represents another limitation.

## Conclusion

The changes in the sagittal parameters measured after bracing showed that TK and LL were significantly decreased and TLJ was slightly increased. Most sagittal plane parameters varied between the standing and sitting positions. Despite the improvement in axial rotation, the effects of changes in the sagittal parameters warrant further investigation. Whether the brace is a victim of its own success is unknown. One of the principles of bracing is to preserve the sagittal profile of the spine, and its effect should be observed through a multidimensional field of view as the multiplanar geometry of the sagittal profile cannot be evaluated using the Cobb angle alone.

## Data Availability

The data contributing to this article may be made available upon request by sending an e-mail to the first author.

## Abbreviations

AIS: Adolescent idiopathic scoliosis
3D: Three-dimensional
CAD: Computer-aided design
CAM: Computer-aided manufacturing
PA: Posterior–anterior
AVR: Apical vertebral rotation
TK: Thoracic kyphosis
LL: Lumbar lordosis
TLJ: Thoracolumbar junction angle
PT: Pelvic tilt
PI: Pelvic incidence
SS: Sacral slope
SVA: Sagittal vertical axis

## References

[1] Weinstein SL, Dolan LA, Wright JG, Dobbs MB (2013). Effects of bracing in adolescents with idiopathic scoliosis. N Engl J Med.

[2] Thompson RM, Hubbard EW, Jo C-H, Virostek D, Karol LA (2017). Brace success is related to curve type in patients with adolescent idiopathic scoliosis. J Bone Joint Surg Am.

[3] Sanders JO, Newton PO, Browne RH, Katz DE, Birch JG, Herring JA (2014). Bracing for idiopathic scoliosis: how many patients require treatment to prevent one surgery?. J Bone Joint Surg Am.

[4] Katz DE, Durrani AA (2001). Factors that influence outcome in bracing large curves in patients with adolescent idiopathic scoliosis. Spine.

[5] Little DG, Song KM, Katz D, Herring JA (2000). Relationship of peak height velocity to other maturity indicators in idiopathic scoliosis in girls. J Bone Joint Surg Am.

[6] van den Bogaart M, van Royen BJ, Haanstra TM, de Kleuver M, Faraj SSA (2019). Predictive factors for brace treatment outcome in adolescent idiopathic scoliosis: a best-evidence synthesis. Eur Spine J.

[7] Emans JB, Kaelin A, Bancel P, Hall JE, Miller ME (1986). The Boston bracing system for idiopathic scoliosis. Follow-up results in 295 patients. Spine.

[8] Romano M, Minozzi S, Bettany-Saltikov J, Zaina F, Chockalingam N, Kotwicki T, Maier-Hennes A, Negrini S (2012). Exercises for adolescent idiopathic scoliosis. Cochrane Database Syst Rev.

[9] Cobetto N, Aubin C-É, Parent S, Barchi S, Turgeon I, Labelle H (2017). 3D correction of AIS in braces designed using CAD/CAM and FEM: a randomized controlled trial. Scoliosis and Spinal Disorders.

[10] Courvoisier A, Drevelle X, Vialle R, Dubousset J, Skalli W (2013). 3D analysis of brace treatment in idiopathic scoliosis. Eur Spine J.

[11] Lebel DE, Al-Aubaidi Z, Shin E-J, Howard A, Zeller R (2013). Three dimensional analysis of brace biomechanical efficacy for patients with AIS. Eur Spine J.

[12] Kwan KYH, Cheung AKP, Koh HY, Cheung KMC (2021). Brace Effectiveness Is Related to 3-Dimensional Plane Parameters in Patients with Adolescent Idiopathic Scoliosis. J Bone Joint Surg Am.

[13] Vergari C, Courtois I, Ebermeyer E, Pietton R, Bouloussa H, Vialle R, Skalli W (2019). Head to pelvis alignment of adolescent idiopathic scoliosis patients both in and out of brace. Eur Spine J.

[14] Lafage V, Schwab F, Vira S, Patel A, Ungar B, Farcy J-P (2011). Spino-pelvic parameters after surgery can be predicted: a preliminary formula and validation of standing alignment. Spine.

[15] Van Royen BJ, De Gast A, Smit TH (2000). Deformity planning for sagittal plane corrective osteotomies of the spine in ankylosing spondylitis. Eur Spine J.

[16] Schwab F, Patel A, Ungar B, Farcy J-P, Lafage V (2010). Adult spinal deformity-postoperative standing imbalance: how much can you tolerate? An overview of key parameters in assessing alignment and planning corrective surgery. Spine.

[17] Vaughn JJ, Schwend RM (2014). Sitting sagittal balance is different from standing balance in children with scoliosis. J Pediatr Orthop.

[18] Lee ES, Ko CW, Suh SW, Kumar S, Kang IK, Yang JH (2014). The effect of age on sagittal plane profile of the lumbar spine according to standing, supine, and various sitting positions. J Orthop Surg Res.

[19] Endo K, Suzuki H, Nishimura H, Tanaka H, Shishido T, Yamamoto K (2012). Sagittal lumbar and pelvic alignment in the standing and sitting positions. J Orthop Sci.

[20] Skalli W, Vergari C, Ebermeyer E, Courtois I, Drevelle X, Kohler R, Abelin-Genevois K, Dubousset J (2017). Early Detection of Progressive Adolescent Idiopathic Scoliosis: A Severity Index. Spine.

[21] Humbert L, De Guise JA, Aubert B, Godbout B, Skalli W (2009). 3D reconstruction of the spine from biplanar X-rays using parametric models based on transversal and longitudinal inferences. Med Eng Phys.

[22] Ilharreborde B, Steffen JS, Nectoux E, Vital JM, Mazda K, Skalli W, Obeid I (2011). Angle measurement reproducibility using EOS three-dimensional reconstructions in adolescent idiopathic scoliosis treated by posterior instrumentation. Spine.

[23] Dubousset J, Charpak G, Skalli W, Kalifa G, Lazennec JY (2007). EOS stereo-radiography system: whole-body simultaneous anteroposterior and lateral radiographs with very low radiation dose. Rev Chir Orthop Reparatrice Appar Mot.

[24] Courvoisier A, Ilharreborde B, Constantinou B, Aubert B, Vialle R, Skalli W (2013). Evaluation of a Three-Dimensional Reconstruction Method of the Rib Cage of Mild Scoliotic Patients. Spine Deform.

[25] Steib J-P, Dumas R, Mitton D, Skalli W (2004). Surgical correction of scoliosis by in situ contouring: a detorsion analysis. Spine.

[26] Almansour H, Pepke W, Bruckner T, Diebo BG, Akbar M. Three-dimensional analysis of initial brace correction in the setting of adolescent idiopathic scoliosis. J Clin Med. 2019;8(11):1804. 10.3390/jcm8111804 PMC6912396 31661811

[27] Rigo M, Weiss H-R (2008). The Chêneau concept of bracing–biomechanical aspects. Stud Health Technol Inform.

[28] Vijvermans V, Fabry G, Nijs J (2004). Factors determining the final outcome of treatment of idiopathic scoliosis with the Boston brace: a longitudinal study. J Pediatr Orthop B.

[29] Landauer F, Wimmer C, Behensky H (2003). Estimating the final outcome of brace treatment for idiopathic thoracic scoliosis at 6-month follow-up. Pediatr Rehabil.

[30] Gepstein R, Leitner Y, Zohar E, Angel I, Shabat S, Pekarsky I, Friesem T, Folman Y, Katz A, Fredman B (2002). Effectiveness of the Charleston bending brace in the treatment of single-curve idiopathic scoliosis. J Pediatr Orthop.

[31] Post M, Verdun S, Roussouly P, Abelin-Genevois K (2019). New sagittal classification of AIS: validation by 3D characterization. Eur Spine J.

[32] Abelin-Genevois K, Sassi D, Verdun S, Roussouly P (2018). Sagittal classification in adolescent idiopathic scoliosis: original description and therapeutic implications. Europ Spine J.

[33] Roussouly P, Berthonnaud E, Dimnet J (2003). Geometrical and mechanical analysis of lumbar lordosis in an asymptomatic population: proposed classification. Rev Chir Orthop Reparatrice Appar Mot.

[34] Roussouly P, Pinheiro-Franco JL (2011). Sagittal parameters of the spine: biomechanical approach. Eur Spine J.

[35] Legaye J, Duval-Beaupère G, Hecquet J, Marty C. Pelvic incidence: a fundamental pelvic parameter for three-dimensional regulation of spinal sagittal curves. Eur Spine J: Official Publication of the European Spine Society, the European Spinal Deformity Society, and the European Section of the Cervical Spine Research Society. 1998;7(2):99–103. 10.1007/s005860050038 PMC3611230 9629932

[36] Mac-Thiong J-M, Labelle H, Berthonnaud E, Betz RR, Roussouly P (2007). Sagittal spinopelvic balance in normal children and adolescents. Eur Spine J.

[37] Zhu W, Liu Z, Sha S, Guo J, Bao H, Xu L, Qiu Y, Zhu Z (2018). Postoperative changes in sagittal spinopelvic alignment in sitting position in adolescents with idiopathic thoracic scoliosis treated with posterior fusion: an initial analysis. J Neurosurg Pediatr.

[38] Clement JL, Pelletier Y, Solla F, Rampal V (2019). Surgical increase in thoracic kyphosis increases unfused lumbar lordosis in selective fusion for thoracic adolescent idiopathic scoliosis. Eur Spine J.

[39] Clement JL, Le Goff L, Oborocianu I, Rosello O, Bertoncelli C, Solla F, Rampal V (2021). Surgical increase in thoracic kyphosis predicts increase of cervical lordosis after thoracic fusion for adolescent idiopathic scoliosis. Eur Spine J.

